# A scoping review of ethics review processes during public health emergencies in Africa

**DOI:** 10.1186/s12910-024-01054-8

**Published:** 2024-05-22

**Authors:** Kingsley Orievulu, Alex Hinga, Busisiwe Nkosi, Nothando Ngwenya, Janet Seeley, Anthony Akanlu, Paulina Tindana, Sassy Molyneux, Samson Kinyanjui, Dorcas Kamuya

**Affiliations:** 1 Africa Health Research Institute, KwaZulu-Natal, Durban, South Africa; 2 School of Nursing and Public Health, University of KwaZulu-Natal, Durban, South Africa; 3 Centre for Africa China Studies, University of Johannesburg, Johannesburg, South Africa; 4 KEMRI Wellcome Trust Research Programme, Kilifi, Kenya; 5 University of Toledo, Ohio, Toledo USA; 6 Global Health and Development Department, London School of Hygiene and Tropical Medicine, London, UK; 7 West Africa Centre for Cell Biology and Infectious Pathogens, University of Ghana, Accra, Ghana; 8 Department of Health Policy, Planning and Management, School of Public Health, College of Health Sciences, University of Ghana, Accra, Ghana; 9 Centre for Tropical Medicine and Pandemic Sciences Institute, Nuffield Department of Medicine, University of Oxford, Oxford, UK

**Keywords:** COVID-19, Ebola virus disease, Ethics review, Pandemic research, Public health emergency, Research ethics committee, Scoping review, Sub-saharan Africa

## Abstract

**Background:**

The COVID-19 pandemic forced governments, multilateral public health organisations and research institutions to undertake research quickly to inform their responses to the pandemic. Most COVID-19-related studies required swift approval, creating ethical and practical challenges for regulatory authorities and researchers. In this paper, we examine the landscape of ethics review processes in Africa during public health emergencies (PHEs).

**Methods:**

We searched four electronic databases (Web of Science, PUBMED, MEDLINE Complete, and CINAHL) to identify articles describing ethics review processes during public health emergencies and/or pandemics. We selected and reviewed those articles that were focused on Africa. We charted the data from the retrieved articles including the authors and year of publication, title, country and disease(s) reference, broad areas of (ethical) consideration, paper type, and approach.

**Results:**

Of an initial 4536 records retrieved, we screened the titles and abstracts of 1491 articles, and identified 72 articles for full review. Nine articles were selected for inclusion. Of these nine articles, five referenced West African countries including Liberia, Guinea and Sierra Leone, and experiences linked to the Ebola virus disease. Two articles focused on South Africa and Kenya, while the other two articles discussed more general experiences and pitfalls of ethics review during PHEs in Africa more broadly. We found no articles published on ethics review processes in Africa before the 2014 Ebola outbreak, and only a few before the COVID-19 outbreak. Although guidelines on protocol review and approval processes for PHEs were more frequently discussed after the 2014 Ebola outbreak, these did not focus on Africa specifically.

**Conclusions:**

There is a gap in the literature about ethics review processes and preparedness within Africa during PHEs. This paper underscores the importance of these processes to inform practices that facilitate timely, context-relevant research that adequately recognises and reinforces human dignity within the quest to advance scientific knowledge about diseases. This is important to improve fast responses to PHEs, reduce mortality and morbidity, and enhance the quality of care before, during, and after pandemics.

## Background

The severe acute respiratory syndrome coronavirus 2 (SARS-CoV-2) accounted for over 624 million infections and over 6.5 million deaths globally in late 2022, and caused the coronavirus disease 2019 (COVID-19) pandemic [1]. The COVID-19 pandemic forced governments, multilateral public health organisations, humanitarian organisations, and academic institutions to undertake research urgently to understand SARS-CoV-2 and respond to the pandemic [2–7]. Research is still needed to generate knowledge on SARS-CoV-2 transmission, to develop vaccines and treatment, and to understand the long-term impacts of infection [3, 8]. This research needs to explore the clinical, biomedical, social, and ethical aspects of COVID-19, including by recognising and incorporating local knowledge systems that are crucial for actualising the scientific and social value of this pursuit.

The conduct of research – especially during a public health emergency (PHE) where the research involves human participants – requires robust regulation against the backdrop of historical injustices and irresponsible practices, and what is often a well-intentioned ‘rush’ for solutions in public health interventions [9–12]. For these reasons, international ethical principles and guidelines are continuously developed, revised, and promoted to ensure that public health research is ethically sound, scientifically relevant, and robust and that human rights are upheld [5, 7, 10, 13–15].

Africa has experienced different infectious disease outbreaks in the last decade, including Ebola virus disease (EVD), HIV/AIDS, yellow fever, Lassa fever, Rift Valley fever, and Mpox, all of which pose serious public health challenges on the continent. Similar to other globally recognised infectious diseases, such as Severe Acute Respiratory Syndrome (SARS) and Middle East Respiratory Syndrome (MERS), these infectious diseases have raised global attention towards research that aims to understand, treat and/or eradicate them [16, 17]. Although humanitarian disasters, social and political instabilities and violent conflicts equally disrupt public health research and interventions, infectious disease outbreaks create even deeper shocks that can further cripple national, regional, and global economies and health systems.

Conducting research during PHEs (used hereafter to designate highly transmittable, infectious and deadly diseases officially designated as epidemics and pandemics) and natural disasters presents particular practical and ethical challenges. Within the context of pandemics such as COVID-19, the role of ethics review committees (ERCs) (used interchangeably in this paper with research ethics committees (RECs)/institutional review boards (IRBs) and national research ethics committees (NRECs)) – hereafter as RECs – is critical [5, 7, 13, 18]. The urgency to understand COVID-19 prompted the development of a plethora of studies to quickly address the emergency, including observational, interventional, clinical, and human challenge studies.

This uncertain situation led to increased efforts to establish or strengthen research collaborations and partnerships as well as community engagement in and for research related purposes [19]. Considerations involved in establishing these partnerships and collaborations included funding priorities, decisions on which science or field of research and which sites or countries to fund. COVID-19 revived the equity concerns discussed within the decolonisation of global health literature and are still discussed and critiqued within and beyond social science [20]. The COVID-19 pandemic created another avenue for old systems of inequitable research practices to become entrenched, but also opened up platforms for debates about equity and equal partnerships in research, especially through prioritising and respecting local knowledge and contextual peculiarities [21, 22].

While the scramble for solutions through research was important, so too are the processes of governing and overseeing the quality, rigour, and ethics of the proposed studies. Pandemic and epidemic contexts – like humanitarian disasters, sociopolitical instability, and violent conflict situations – cause complex and difficult dilemmas within the health system, and negatively impact efforts to establish and implement interventions. These complexities may be wide ranging in the quest for scientific breakthroughs. Maintaining the primacy of human rights and dignity in these situations can be challenging.

Documented ethical issues in conducting research during PHEs include preparing RECs for accelerated review of studies, for instance through the establishment of ad hoc committees [3, 6, 23–30]; ensuring appropriate research designs for scientific validity, social value and fair selection of participants [31]; promoting inclusive and adequate stakeholder engagement and informed consent processes [2, 32–34]; dealing with the specific ethical conundrum of clinical trials and human challenge studies during emergencies [35–41]; supporting appropriate data collection, storage and future use, including those relating to children [33, 42–45]; and maintaining mechanisms for ethics review whether in person or virtual [40, 46–51]. However, few studies specifically consider ethics review processes, procedures and governance frameworks for epidemics and public health emergencies in Africa [52].

In this paper, we examine the landscape of ethics review processes in Africa during PHEs. Our objectives were to identify the context and content (where possible) of such processes and identify emerging issues. We also aimed to identify gaps in the literature on this topic in and for Africa.

## Methods

We conducted a scoping review to explore existing literature reporting on ethical review processes and how they were structured, articulated, and managed in Africa within the context of PHEs. This is an important area in research given the challenges that PHEs could pose to ethics review processes supporting the timely conduct of scientifically rigorous and ethically sound research. We chose to undertake a scoping review because very little was documented about ethics review processes during PHEs in Africa. Scoping reviews are often exploratory [53], flexible and can combine sources based on both qualitative and quantitative analysis. Scoping reviews are descriptive and can be used to rapidly explore the field, identify research gaps [53, 54].

Based on our interest in ethics review processes in Africa and their importance for effective, robust, and ethical governance of research during PHEs, this scoping review was important in assessing the mechanisms for ethics review in Africa, and in describing their processes for undertaking protocol reviews during PHEs. Arksey and O’Malley identify five stages of the methodological framework for scoping reviews: identifying the research question; identifying relevant studies; study selection; charting the data; and collating, summarising, and reporting the results [54]. We adapt this framework for our paper, outlining the research question(s), the search strategy, study screening and selection, data analysis, and findings.

### Research question(s)

The broad question we address in this review is: What are the emerging ethical and practical issues within ethics review processes, frameworks, and procedures during PHEs? This is followed by two sub-questions, namely: What is the landscape of ethical review processes during PHEs? And how have ethical review processes been structured and executed during PHEs?

### Search Strategy

We searched four electronic databases: Web of Science, PUBMED, MEDLINE Complete, and CINAHL (12 August − 12 September 2021, and 30 March 2023). Our main focus was on articles showing African experiences of PHEs. We followed Arksey and O’Malley’s scoping review guidance and framework, keeping the search terms flexible enough to accommodate as many articles as possible within the broader scope of the review [54]. We considered all materials available in these databases including unpublished work (like pre-prints), reports and commentaries.

We used the Preferred Reporting Items for Systematic Reviews and Meta-Analyses extension for Scoping Reviews (PRISMA-ScR) to guide the selection process [55]. Table 1 shows the search terms used, while Table 2 indicates our inclusion and exclusion criteria.

**Table 1:** Search Strategy

Database(s)	Search Keywords
PubMed	((ethic*[Title/Abstract]) AND (((governance[Title/Abstract] OR regulat*[Title/Abstract] OR oversight[Title/Abstract] OR codes[Title/Abstract] OR guidelines[Title/Abstract]) OR (ethical review OR ethics committees[MeSH Terms])) OR (“ethical review“[Title/Abstract]))) AND (pandemic*[Title/Abstract] OR “public health emergenc*“[Title/Abstract] OR disaster[Title/Abstract] OR COVID-19[Title/Abstract])
Web of Science	
Medline Complete	
CINAHL	

**Table 2:** Inclusion and Exclusion Criteria

We included articles that showed: • RECs coordination efforts, strategies, and processes towards reviewing research protocols during pandemics and PHEs. • Recommendations about the role of RECs and actions to facilitate the review of research protocols during pandemics and PHEs. • Guidelines and recommendations on the conduct of ethics review procedures or processes during pandemics (Ebola, COVID-19, Zika etc.). • Bioethics arguments – in commentaries, opinion pieces, reviews – focusing on RECs procedures and/or oversight functions/roles/processes in or for the review of protocols for and during pandemics/PHEs. • Experiences and analysis of RECs during PHEs in, across and about, Africa.
We excluded articles that: • Focus too narrowly on ethical considerations and issues in pandemics from a Bioethical analytic process; articles not grounded on the processes or procedures of ethics review committees. • Not focused on the review of research protocols developed during PHEs. • Report of post-pandemic research and or ethical issues in general. • Narrowly focus on pandemic response or interventions – not specifically linked to research activities, research ethics, IRB/REC processes/oversight/procedures or research protocol review. • Narrowly focused on the external effects or impacts of COVID-19 or any other pandemic/disaster (on populations/economies/health outcomes) without exploring IRB processes/procedures or IRB procedures pertaining to protocol reviews. • Focus on natural disasters and post-Natural disaster interventions and research activities in affected areas. • Not published in English. • Not fully accessible for full review. • Not focused on, or make clear references to, Africa (during the final review).

### Study screening and selection

We imported all retrieved entries into Rayyan, an online systematic review management tool. KO and AH used Rayyan to automatically identify and exclude duplicates and conducted an initial screening by title, abstract, and full content review. This was followed by a second screening in which only articles from, reporting on, or referencing, the African context were included. Where there were disagreements, senior team members BN, NN, DK, and JS provided adjudication. We exported the data from Rayyan into Microsoft Excel and classified them by their Rayyan identification numbers, article title, author name, year of publication, country reference, disease reference, broad topic covered, paper type, and approach. These were initially selected for the second phase of the review and screened using the inclusion criteria set out in Table 2. The actual studies included in the study were largely peer-reviewed publications, reflection/discussion papers and essays – all of which are allowed within the context of a scoping review [53, 54].

### Data analysis

In line with Arksey and O’Malley [54] and Peters et al. [53], we conducted a descriptive thematic analysis. The broad thematic areas covered included the country or regional context within which ethics review processes were identified; the description of ethics review processes and regulatory frameworks identified or mentioned in the articles; and the considerations identified by RECs during review processes, ranging from study design to informed consent, collaborative partnerships, and engagement. These themes were drawn from our review objectives and emphasised based on the level of attention afforded them by the papers reviewed.

**Table 3:** Table of Reviewed Articles

Authors + Year	Title	Country Reference	Disease Reference	Topic Areas addressed	Paper type	Approach
Alirol, E et al. (2017) [56].	Ethics review of studies during public health emergencies - the experience of the WHO ethics review committee during the Ebola virus disease epidemic.	Unspecified	Ebola	Considerations in PHE study protocol reviews	Discussion paper	Mixed Methods
De-Crop, Maaike et al. (2016) [57].	Multiple ethical review in North‒South collaborative research: the experience of the Ebola-Tx trial in Guinea.	Guinea	Ebola	Joint (double) ethics review	Discussion paper	Qualitative
de Vries, J. et al. (2020) [58].	Research on COVID-19 in South Africa: Guiding principles for informed consent	South Africa	COVID-19	Informed Consent	Discussion paper	Qualitative
Saxena, A. et al. (2019) [23].	Ethics preparedness: facilitating ethics review during outbreaks - recommendations from an expert panel	Unspecified	Ebola and PHEs	Ethics preparedness and considerations for PHEs	Discussion paper	Qualitative
Schopper, D et al. (2017) [59].	Research Ethics Governance in Times of Ebola	Unspecified	Ebola	Ethics review processes	Discussion paper	Qualitative
Bain, L. E. et al. (2018) [60].	Research Ethics Committees (RECs) and epidemic response in low- and middle-income countries	Unspecified	Ebola and Zika	REC considerations in ethics review frameworks development	Essay	Qualitative
Doe-Anderson, J. et al. (2016) [61].	Beating the odds: Successful establishment of a Phase II/III clinical research trial in resource-poor Liberia during the largest-ever Ebola outbreak	Liberia	Ebola	Clinical trials	Discussion paper	Qualitative
Folayan, M. O. et al. (2021) [62].	Considerations for stakeholder engagement and COVID-19 related clinical trials’ conduct in sub-Saharan Africa	Unspecified	COVID-19	Stakeholder-engagement for Clinical trials	Original research	Qualitative
Hinga et al. (2022) [52]	Pandemic preparedness and responsiveness of research review committees: lessons from review of COVID-19 protocols at KEMRI Wellcome Trust Research Programme in Kenya	Kenya	COVID-19	Research and ethics review processes	Original research	Mixed methods

### Findings

**Fig. 1:**
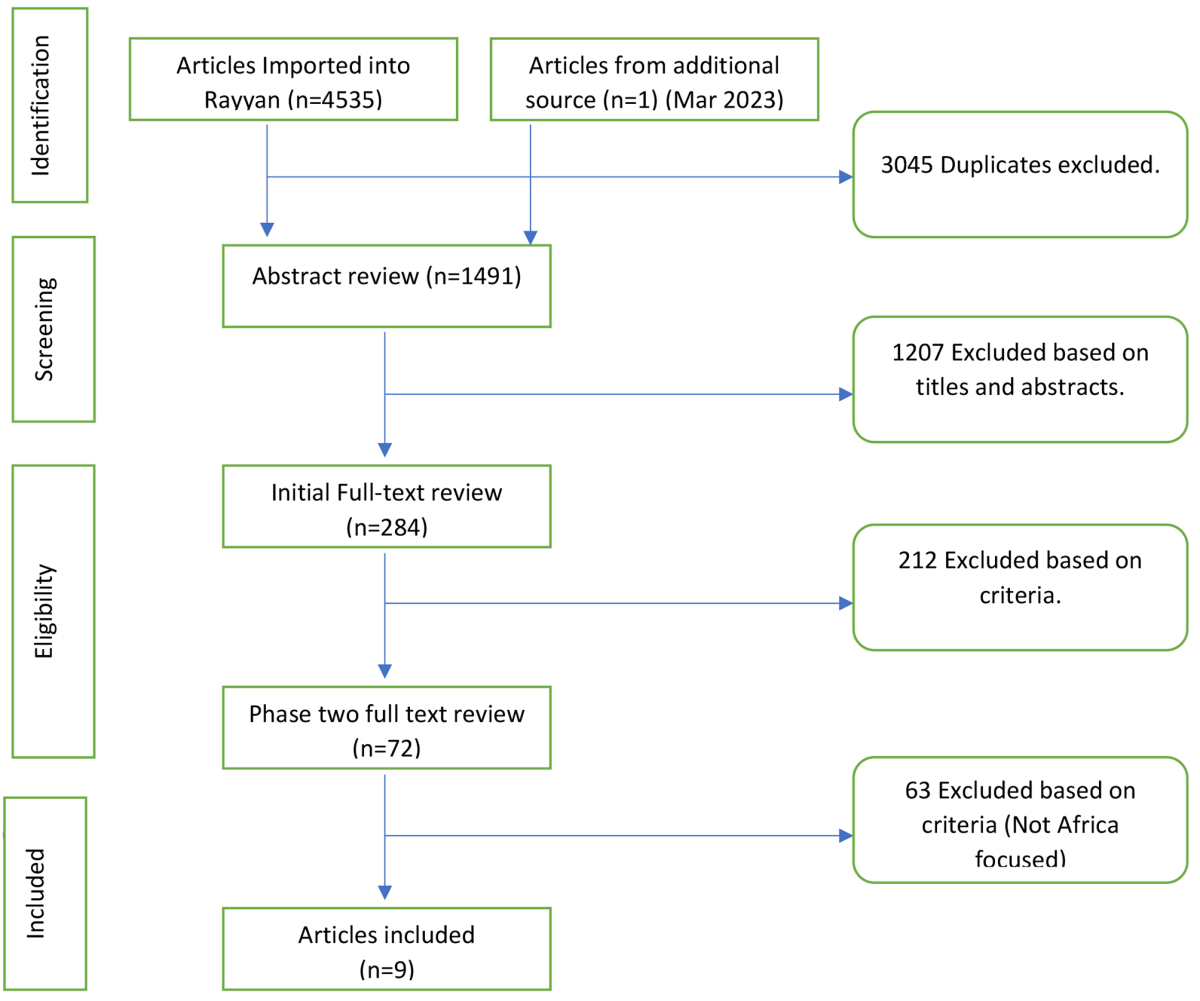
PRISMA Flowchart of Study selection

We located 4536 potential papers from our initial search. After removing 3045 duplicates, they were reduced to 1491, which we screened. A further 1207 were excluded after reviewing titles and abstracts for relevance to the review question(s). We then conducted two rounds of full-text review of the remaining 284 articles, first excluding 212 articles that did not meet our criteria, and then excluding 63 of the remaining 72 because they were not about Africa. Nine articles were included in the final review. Figure 1 shows the PRISMA flowchart.

As shown in Table 3, most (6/9) of the included articles are discussion papers published in peer-reviewed journals [23, 56–59, 61]. Two original research articles were included: a qualitative methods paper [62] and a mixed method paper with a strong qualitative element [52]. Six articles covered issues related to ethics review and EVD considerations [23, 56, 57, 59–61]; three covered issues linked to COVID-19, including (informed) consent [58], stakeholder engagement [62] and research and ethics review [52]; three commented on virtual modalities for review (meetings), consultations or obtaining consent [23, 52, 58]; and five described review timelines during public health disasters [52, 57, 59–61].

Most articles were drawn from or referenced West African countries including Liberia, Guinea, and Sierra Leone, either individually ( [57, 61]) or as a collective [23, 56, 59]. These were the countries most affected by EVD between 2014 and 2016. One article drew on South African experience [58] and another on Kenyan experience [52] during the COVID-19 pandemic. The remaining two articles reference Africa broadly [62] or extrapolated from an African experience to make comments about ethics review processes during PHEs across low- and middle-income countries [60].

### Thematic areas in the African ethics review process landscape

In this section, we describe two broad thematic areas emerging from the scoping review: A) The processes, procedures and frameworks established or drawn upon by RECs to facilitate timely and robust reviews under PHEs. Process-related sub-themes describe the preparatory and challenging components of these REC processes. B) The considerations that were highlighted or flagged by RECs or the articles pertaining to research being conceptualised, planned for, or conducted in, the context of PHEs. The sub-themes described under these considerations emphasise ideas around the nature of proposed studies, including important ethical and practical concerns within PHE-related research.

### A) Processes, procedures and frameworks for ethics review during PHE

#### Preparing for (accelerated and robust) ethical reviews during outbreaks

The reviewed articles describe different aspects of processes, procedures, and frameworks set up or implemented during PHEs. Five studies describe experiences of establishing or reviewing clinical trial(s) or intervention studies and some the procedural aspects of ethics review [52, 56, 57, 59, 61]. Three articles emphasise preparing review bodies [23, 52] or their members in LMICs [60] for reviewing study proposals during outbreaks through skills audit and training. Saxena et al. [23] discuss preparing committees as a priority outcome of the 2018 workshop organised between the World Health Organisation Global Health Ethics Team and the African coalition for Epidemic Research, Response and Training (ALERRT) [23]. The articles describe the need for accelerating review processes, audits to identify and address competency gaps among REC members through training, and approaches to review studies in the event of future (and possibly deadly) infectious disease outbreaks [23, 52, 60].

#### Regulatory and procedural issues for accelerated reviews

The reviewed articles show some of the important steps undertaken to facilitate accelerated reviews. These include the World Health Organisation Ethics Research Committee (WHO-ERC) development of and reliance on Standard Operating Procedures (SOP) – or Rules of Procedures – for accelerated review during emergency periods [23, 56]; establishing the protocol review subcommittee; and ensuing training for specialised subject-area reviews. Alirol and colleagues [56] highlighted the importance of the WHO-ERC Rules of Procedure, noting that “the EVD outbreak was the first-time accelerated review was implemented” [56]. These rules provided the framework for sensitising WHO-ERC members on the plans by the WHO to rapidly review Ebola disease-related studies. The established WHO-ERC subcommittee was populated by volunteers recruited on short notice. The newly established system designed to accelerate reviews and protocol assessment was characterised by regular monthly meetings (face to face or teleconferences) and task allocations [56].

Saxena et al. highlighted another priority issue for preparedness: pre-review of generic (non-context specific) research protocols by RECs [23]. The generic protocols were to be developed outside of a period of infectious disease outbreaks, facilitating applications for review early during an outbreak. These protocols are easier to review by REC and can be adapted, thus increasing speed without compromising quality [23]. Reflecting on their experiences of submitting research protocols for review during the COVID-19 pandemic, researchers in Kenya supported the idea of pre-reviewing generic protocols in non-emergency times to accelerate research review and implementation during public health emergencies [52].

#### Membership composition of review committees

Within the EVD context, Alirol and colleagues [56] highlight that setting up the review of intervention and preventative studies, especially clinical trials of therapeutic products, required the WHO-ERC to draw on guidelines of the Council for International Organisations of Medical Sciences (CIOMS) concerning conducting research with human participants, especially during disasters and public health emergencies [56]. This WHO-ERC was constituted as a 27-member committee consisting of experts “in clinical research, drug development, social sciences, [and] legal affairs”, including “a lay member” [56]. “Between 6 and 8 members are from Geneva-based universities or international organisations” [56]. Schopper and colleagues [59] also highlight that the Medecins Sans Frontieres Ethics Review Board (MSF-ERB) contributed and worked with other institutions towards the design and review of (intervention) study protocols, including clinical trials. This was in line with the International Health Regulation’s (IHR) declaration about EVD and the WHO permission for the ethical use of unregistered interventions in the treatment of Ebola patients [59].

#### Multiple review processes

Six articles describe processes for undertaking double or multiple ethics reviews within collaborative/partnership research which is jointly funded [23, 52, 57, 59–61]. For example, the Ebola-Tx clinical trials study funded by a European Union grant was sponsored by the Institute of Tropical Medicine (ITM) and implemented at the MSF Ebola Treatment Centres (ETC) in Guinea [57]. De-Crop et al. [57] highlight that in this study, the initial process involved forming a research consortium comprising 17 institutions led by the ITM. Consequently, the study protocol had to go to multiple RECs from the study country (Guinea) and the sponsor country (Belgium) and to the institutional committees of collaborating institutions such as ITM, MSF, WHO, and LSHTM. Although other collaborators did not demand the submission of the protocol for assessment, the scientific Commission of the National Ebola Coordination in Guinea considered the scientific rationale of the study before issuing an initial approval for the study in Guinea [57]. The requirements for these review processes included the protocol (to be reviewed by coordinators from Belgium and Guinea), informed consent documents, a no-fault insurance certificate, CVs of the scientific coordinator and country PI, and case report forms [57]. Although much of the documentation required was similar, there were some differences in the content and modalities of submission across institutions, essentially resulting in multiple ethics review process, with implications for the timing of the trial.

Similarly, the Partnership for Research on Ebola Vaccines in Liberia (*PREVAIL*) was a collaboration between the USA and the Liberian government to establish and implement clinical trials under the EVD context in Liberia [61]. Reviews were therefore required from the different country bodies of the funders and country where the trial was implemented. Doe-Anderson and colleagues [61] highlighted that the protocol was submitted to two regulatory authorities in each partner country: the Federal Drug Authority and the IRB of the National Cancer Institute (NCI) at the National Institutes of Health (NIH) in the United States and the Liberian Medicines and Health Products Regulatory Authority (LMHRA) and National Research Ethics Board (NREB) [61].

Schopper et al. also report elements of multiple reviews for studies reviewed by the MSF-ERB and other institutions, including Oxford University and London School of Hygiene and Tropical Medicine [59]. They note: “Of the 27 protocols reviewed by the MSF-ERB, 11 were in addition reviewed by a national EC only, while 7 were reviewed by a national EC and one or several ECs/IRBs from other international institutions or academic centres” [59](p.52–54).

The multiple review processes undertaken for PHE studies are often intended to support partners, funders and institutions involved in collaborations to achieve the rapid but robust protocol reviews before their implementation [23].

#### Bottlenecks around multiple reviews

Five articles highlight drawbacks associated with multiple reviews, especially within a drive to achieve expeditious and scientifically robust reviews during outbreaks [23, 52, 57, 59, 60]. Protocol submission requests, specific REC comments, revisions, and resubmissions – requiring researchers’ replies to specific REC queries – often impacted timelines for the review process and study implementation [57, 59, 60]. The varying capacities and processes of different review committees, in terms of review turnaround times, for example, were highlighted as a major challenge for multiple reviews since researchers had to respond to reviewers’ comments on different versions of submitted protocols [52]. Although multiple review processes aim to ensure robust ethical standards and quality review, these did not insulate the process from possible debilitating complexities, hence the emphasis on the adoption of joint reviews and coordination to harmonise the process and circumvent some of these inherent complexities [23, 59, 60].

#### Guidance documents for outbreak reviews

Studies also reference different international and national guidelines, proposals, consultation outcomes, recommendations, and frameworks, broadly governing the design and implementation of the studies. This includes guidance on ethics review processes and procedures for the studies being reviewed, whether clinical trials, experimental studies, intervention studies or other types of studies. For example, the Ebola-Tx trial processes relied on the 2014 WHO “consultation on vaccines and therapies” [57]. This consultation resulted in a consensus on the imperative for the rapid development of study protocols for effectively testing vaccines and therapies that demonstrate promise to be used for interventions. To facilitate trust in this process, multiple institutional reviews and harmonising such processes across the different ethics committees were viewed as crucial [57].

De Vries and colleagues draw on national (South African) and international guidelines to discuss important questions and applications of informed consent while designing, reviewing and implementing COVID-19-related studies and interventions [58]. Folayan and colleagues focused on elements of the “Good Participatory Practices for Emergency Pathogens” (GPP-EP) to discuss the centrality of stakeholder engagement in designing and implementing COVID-19 clinical trials in SSA [62]. Hinga et al. highlighted the development of institutional and national-level guidelines for review of research protocols during the COVID-19 pandemic in Kenya, which included guidelines for protecting participants and research staff from infection during data collection and guidance on remote submission and review of protocols [52]. The fundamental role of, and reliance on, ethical and practical documentation guidelines is arguably considered vital to the efficiency and transparency of ethics review processes. Adapting these to unique outbreak circumstances and contexts is a major aspect of the review process.

#### Review timelines

Timelines for the review and approval of protocols varied within the different reviewed articles. In the case of the WHO-ERC, an average of six working days was reported for the WHO-ERC teams to review protocols submitted under the context of the EVD [56]. This was different from the reviews conducted by the MSF-ERB, which reported over 30 days between initial request and final approval [59]. In the latter, there was an initial average response time of 12.4 days from initial submission to replies from the investigator. Although this timing reduced to 1–4 days, the influx of more protocols increased the MSF-ERB workload [59]. In Kenya, the review of research protocols during the COVID-19 pandemic was faster than during the pre-pandemic period. However, internally set targets for review turnaround times during the pandemic were generally not met; there was a 5-day delay by the national review committee in providing initial feedback on new research protocols [52]. The PREVAIL study took less than 30 days to obtain all required approvals after intentional strides to address the concerns raised around conducting vaccine trials on people [61], and the Ebola Tx Trial study took 55 days [57]. Bain and colleagues critiqued the conventional system of ethics reviews, which took between 24 and 44 days, as counterproductive to the goal of gaining an understanding of new infectious diseases [60].

### B) Considerations identified during review processes

#### Appropriateness of the proposed study design

Five articles discuss the appropriateness of different study designs in the context of PHEs [56, 57, 59–61]. The study design was reviewed in relation to the need for scientific validity, social value, and minimising risk while maximising benefits during PHEs and infectious disease outbreaks [56, 60].

Randomisation in clinical trials and experimental intervention studies for EVD was highlighted as particularly challenging, with RECs proposing a change of design for all participants to receive the experimental intervention treatment [56, 57, 59, 61]. For the COVID-19 pandemic, RECs highlighted the need to account for loss to follow-up while calculating study power given the significant disruption and uncertainty [52]. The PREVAIL Vaccine trial considered both a RCT and ring design but chose the RCT design because it provided “… the greatest likelihood of providing more definitive results, and could potentially lead to rapid licensure and availability of effective vaccines” [61]. The MSF-ERB determined a priori to use the ring design as community engagement consultations (through MSF) revealed that randomisation was unacceptable, as it represented a “lottery system” for receiving the intervention (in the clinical trial) despite high Ebola mortality in the community [57]. For the WHO-ERC, protocols that provided strong arguments for the benefit-risk ratio assumption were accepted if they came from the Ebola-affected countries [56].

- *Formative research to inform the development of protocols and appropriate research designs during PHEs.*

Linked to ensuring the appropriateness of research design to context, four papers noted the importance of formative research during PHEs [59–62]. Formative research allows consideration of crucial and sensitive components of the social contexts, cultural norms and practices and potential misgivings, fears and sensitivities to be considered in the design and conduct of clinical trials [60, 62].The feasibility of undertaking formative research was raised, linked to safety as well as time for approvals [59]. Nevertheless, Bain and colleagues [60] emphasised the importance of formative research during emergencies, especially to inform randomised trials. They promote the use of rapid anthropological research methods during disasters as warranting the attention of RECs, the response team, and researchers.

- *Study population and involving vulnerable populations in research and intervention.*

The study population within protocols under review during PHEs was discussed in four articles [56, 57, 59, 61]. RECs definitions of risk and benefits, and how consent should be obtained, influenced their different views regarding the inclusion or exclusion of vulnerable populations [57, 59]. One REC suggested that researchers should provide alternative methods of participation for individuals without smartphones to prevent unfair exclusion of participants during the COVID-19 pandemic [52]. The PREVAIL study excluded pregnant women, lactating mothers, and children because of the inadequacy of safety data [61]. The WHO and MSF RECs however emphasised the importance of including pregnant women, children and unaccompanied minors unless their exclusion was justified based on data demonstrating greater risk than standard of care [56, 59]. Although neither REC rejected protocols that excluded these vulnerable populations, these examples highlight essential ethical study design considerations during PHEs.

#### Addressing informed consent in study protocols

Informed consent was referenced in most of the articles reviewed [56–60, 62], with discussions on delayed, proxy and waived consent [56, 58]. The WHO-ERC waived consent for two protocols aimed at retrieving anonymised information from patient records [56].

Some papers emphasised the need to guard against scenarios of situational coercion in participants’ recruitment, including scenarios where a third party – husbands, parents, or local chiefs – may influence an individual’s participation [56, 62]. Contexts of deadly outbreaks such as the EVD can facilitate scenarios where people are tacitly coerced to participate in a study without adequate information and informed consent [56]. The WHO-ERC thus emphasised ensuring that information documents are simplified in the language of participants and well explained [56]. In the Ebola-Tx study, the REC required the researchers to provide clarity around considerations of consent related to minors and unaccompanied minors [57]. The consent of parents in this context (for minors) and other third party actors, such as local chiefs, is designed to protect potential participants but can lead to coerced consent in communities and families [62]. The training and capacitation of researchers, implementers, and REC members were therefore emphasised [60], and the involvement of the community in developing informed consent documents recommended [56, 58, 62].

#### Prioritising stakeholder engagement

Four articles referenced the importance of stakeholder engagement, consultation, and involvement in the process of designing and implementing research studies [58, 60–62]. Stakeholder engagement is a component of the “Good Participatory Practices for Emergency Pathogens (GPP-EP)” [62]. De Vries and colleagues [58] reflected on the importance of community engagement using new and conventional media (including social media, TV, radio, and newspapers) to facilitate information sharing and communicate findings, especially where in-person contact was difficult. The authors recommended that ethics review processes impress on researchers to ensure that “community and public engagement [are] genuine and robust, long term and include a plan for post-pandemic communication of research results and plans for long term sample and data storage” [58].

The PREVAIL study used the concept of social mobilisation and communication (SMC) to emphasise multistakeholder engagement in the planning, recruitment for, and implementation of clinical trials [61]. This aimed to better understand local perceptions of and attitudes towards the EVD against the backdrop of views that EVD was man-made, externally curated, and transmitted to populations through clinical trials [61]. Indeed, deep-seated myths, mistrust, and suspicion about interventions, the government and the disease(s) require not only education but also advocacy and consultations to manage (and subsequently implement) research without widespread disapprovals from local populations [58–62]. Study protocols therefore had to reflect strategies for stakeholder engagement, and RECs had to emphasise and request that study designers – PIs – and sponsors address these issues during review processes [58, 60].

#### Demonstrating collaborative partnerships within study protocols

Three articles showed the need for protocols submitted for review during PHEs to demonstrate equitable collaboration and partnerships between external and local researchers [56, 60, 61]. Collaborating in research during PHE has important implications for ethics review including the need for proposed studies to truly reflect the local context of research and interventions. However, a number of factors can contribute to a lack of collaboration between local and external researchers including marginalisation of local actors and researchers [60]. Thus, the WHO-ERC requested protocols to clarify the involvement of (and nature of collaboration with) local scientists and local actors for better contextual analysis and engagement with communities and people within the context of EVD research and interventions [56]. The emphasis by the WHO-ERC emanates from documented reports of heightened levels of mistrust within some African countries about externally funded research linked to infectious diseases outbreaks [59–61]. Such mistrust is traced to historical injustices and experiences such as polio-related side effects in Nigeria and other issues such as local attitudes towards blood and linked misgivings about the collection and storage of people’s blood samples [59–61].

This is relevant considering the critique of the poorly established nature of collaborative frameworks and partnerships between researchers and institutions from donor and local contexts, especially as regards the management and recognition of ethics approvals [60]. While double or multiple, but harmonised, ethics review of multiple site study protocols is generally acceptable [56, 57, 59, 60], situations of unequal collaborative activities elongate the review process. This makes it largely difficult for timely review because the externally imposed institutional guidelines and processes lack adequate relevance in the context of research implementation, leading to missed research opportunities [60].

#### Data/Sample sharing and future use

Data and sample collection, storage and sharing remain very sensitive issues in the context of research and interventions during PHEs. These often challenge the concept of equitable partnerships in research collaborations between institutions in the Global North and those in Africa, and accounts for the attention that research review processes pay to them. In this review, these issues were predominantly referenced by three articles [23, 58, 59]. Studies found it unethical to impose a blanket ban on data and sample sharing but proposed that critical ethical questions must be raised. It was proposed that RECs could establish modalities that can be used to review protocols in the area of data sharing. On the one hand, this would entail the requesting research sponsors and PIs to submit preliminary data and sampling sharing plans on how data generated will be shared. On the other hand, these applicants can be requested to submit full-data sharing plans when resubmitting their (now full) application [23].

Accordingly, de Vries et al. argue that RECs must establish guidelines that clearly define the types of research data that can be used in the context of imperfect informed consent – bearing in mind that there is a high likelihood of such situations during disease outbreaks [58]. While debates remain about how ethical (or not) it is to use samples collected during the COVID-19 pandemic for broad population genomic studies or to interrogate questions completely unrelated to the condition [58] (p. 638), the studies highlighted the importance of RECs to guide the modalities for data sharing and use through engagements with PIs and study sponsors within the process of reviewing proposals during pandemics. The process would ensure that researchers clearly outline plans and justifications for storage, sharing and future use of data and/or samples in their ethics clearance application [56, 58]. It would also ensure a clear description of whether samples and data will be stored and shared, who they will be shared with (with or without restrictions), and what they will be used for in the future [56, 58].

In the case of blood samples,, Schopper and colleagues contend that the ethics review process must be used to ensure that protocols explicitly indicate if blood samples collected during the research study will be destroyed or stored for future use [59], and that this information must be clear in information sheets and consent forms and the patients/participants must explicitly indicate their choice/decision. For example, in all the studies that they reviewed, the WHO-ERC explicitly requested for clarifications on sample and data ownership, data sharing policy, processes for decisions on future use of samples and appropriate participant information [56]. Nevertheless, in view of the urgency of the EVD, the WHO-ERC approved studies where researchers demonstrated commitment to put appropriate agreements/processes in place [56].

The articles reviewed provided several recommendations pertaining to ensuring preparedness, efficiency and effectiveness of ethics review processes. Central among these was the need for the harmonisation of review processes through mechanisms such as joint review committees [57] – with representatives of individual ethics committees [56] – in cases of double, multiple and/or multi-site reviews [23, 56, 59]. This mechanism would enhance direct dialogue between the different ethics committees and reduce duplication or contradictory reviews [23, 56, 57, 59]. Capacity building, upskilling, and training for REC members, partner institutions, and local investigators was also recommended; while the need to emphasise or institute the use of generic protocols that could be considered in the event of emergencies was buttressed to reduce time spent on long arduous processes [23, 56, 59]. Finally, authors of the papers reviewed recommended the need to simplify ethics review guidelines, clarify and agree on terminologies used in generic protocols [23, 60], flexibility with REC processes, and the need for governments and donors to provide adequate resources which would allow for more anthropological research processes [59].

## Discussion

This review explored the landscape of ethics review processes, procedures, and frameworks in Africa. We identified that within the African context, there is a gap in the published literature on research ethics review processes during PHEs, including protocol review processes, preparedness, and priority-setting. While the Ebola virus disease provided the basis for more active engagement with the issue of oversight and governance for ethics review processes, little has been published on ethics review processes, and frameworks during PHEs within the African landscape. This is an important area of interest because there are studies, commentaries and opinion pieces on intervention or response strategies and activities in the event of PHEs such as the recent COVID-19 pandemic or disasters in general; and for research to be undertaken, it must first be approved by a REC.

### Nuanced reliance of RECs on international guidelines for research during PHEs

We found that the ethics review processes relied on, and referenced, existing international ethical guidelines and principles for managing research, including during PHEs [5, 14, 15]. Most of the reviewed articles demonstrated that the processes undertaken, established, or adopted within the context of the PHEs within which they emerged, were rooted in these international principles relevant to the conduct of pandemic research. This is in line with different global studies where emphasis has been placed on international guidelines and guiding principles on ethical conduct of research processes [16, 25, 63, 64].

Bain and colleagues, however, critiqued the pervasiveness of the existing international guidelines on the basis of the contextual appropriateness of some of these guidelines [60]. This is especially important in terms of their potential negative impacts on timely and swift but robust and context-sensitive ethics review processes for study protocols during PHEs or disasters. They note that without quick turnaround review times, much could be lost with regards to the knowledge that could be gained from data collected at crucial – not redundant – contexts of the infectious disease outbreak. These issues point to what many commentaries perceive as a top-down approach to pandemic preparedness that have potential ramifications for ethics review processes. One might describe this as a hegemonic dependence on international guidelines that are not locally developed or truly reflective of local content and context. Such outlooks echo aspects of decolonisation literature that lay emphasis on privileging internationally imposed systems, including those of knowledge production, within strides to prepare for and manage research processes during PHEs in Africa [20–22, 65].

However, while these international frameworks and guidelines provided the impetus and inspiration for many of the structures laid out within these processes during PHEs, they were not the only sources of guidance. Due to the importance of context-specificity, different country and REC contexts, the REC membership had to rely on their uniqueness to develop appropriate processes (or adapt existing ones) and guidelines to address their needs. For example, despite the importance of the Good Clinical Practice to South Africa, the country has national principles and legislations guiding the conduct of research involving human subject which are adapted to particular situations such as the COVID-19 context (these include the National Health Act No. 61 of 2003; the National Department of Health Guidelines on Ethics in Health Research, and South Africa Health Products Regulatory Authority (SAPHRA) South Africa Good Clinical Practice.) Thus, while international guidelines remained quite central to the mechanisation of ethics review processes and frameworks within Africa, reliance on them has been nuanced, and they have been – are being or have to be – applied contextually.

### Similarity of process(es) for PHEs ethical research governance

Additionally, the studies reviewed share similarities in terms of processes set in motion or recommended to be put in place to facilitate accelerated review in current PHEs or in preparation for future experiences. The need for rapid, expedited ethics review of study protocols to facilitate a speedy investigation into disease pathogenesis as well as therapeutics and interventions required to curb the spread and impacts of the diseases was a cross-cutting theme in the papers reviewed [60]. A central idea here entailed constituting ex-temporal committees or processes of review, including joint and multiple reviews [23, 56] to reduce the propensity for long and duplicated reviews [3, 4]. Such a framework, which allows for representatives of individual RECs to form part of the ad hoc committee, allows for a central review and approval process for which the final decision will be reported back via the same channel, thus reducing the time for the review of articles.

This process contributes to crucial recommendations, such as the development or amendment of the SOP pertaining to the conduct of research, and considerations of the context-specificity of PHEs. Other recommendations include enhancing flexibility for how ethics review processes work, the manner of coordination and harmonisation required in cases of multisite studies, joint reviews, and collaborative activities to strengthen the review processes, robustness, and speed of reviews [3, 24, 48]. The idea of establishing ad hoc committees is not universally endorsed, as some contend that ad hoc committee processes unnecessarily impede the desired expedition of reviews and defeats the purpose for which it was proposed or recommended [30]. Indeed, steps undertaken to expedite the review process do not often yield the intended result – a point confirmed by De Crop and colleagues’ experience of the Ebola Tx Trial study [57].

### Relevance of the content of REC reviews for ethical principles during PHEs research

Interrogating the content of the ethics review processes, we also found that issues such as study design, the nature of informed consent – especially how information is communicated, and consent obtained – and the importance of stakeholder engagement and collaborative partnerships were high on the agenda of most of the Africa-specific literature reviewed. This is in line with the requirements emphasised within COVID-19 institutional research contexts and perspectives, and beyond. A COVID-19 research review intervention in New Jersey (USA) ensured that proposals that lacked clear research questions, methodology and research designs were rejected (internally); they were therefore not allowed to proceed for REC approval [66]. At the core of this emphasis is the need for study protocols, and research review processes to strengthen, not minimise, important ethical principles – from respect for persons, the imperative to do no harm, maximise benefit and minimise risks under beneficence, to demonstrating fairness and justice particularly in the selection of study participants – during infectious disease outbreaks [66–68]. These findings are also captured in the broader literature on the implementation of research during pandemics and disasters [47, 69, 70], as well as the ethical considerations that researchers and sponsors must bear in mind in their design and implementation of studies that involve human subjects [4, 43, 65], especially those from vulnerable communities and populations [70].

This emphasis reiterates the push against prioritising the pursuit of scientific knowledge above respect for human rights, dignity, and autonomy, especially within the frame of respecting participants and their autonomy, the processes of seeking consent, and the information contained in the consent forms during PHEs [7, 9, 14, 67]. With regards to informed and voluntary consent and participation, the role of ethics review processes to institutionalise human rights and dignity is revealed within the contents of REC recommendations and areas of emphasis in the articles in our review. This aligns with the dominant literature and debates about what consent is and how it should be obtained, how much information should be shared about interventions (proven and unproven), risks and benefits, and who can give consent during PHEs research [5, 15, 16, 63, 67, 71]. These study designs, methodological and other ethical considerations serve to strengthen the research process against the reoccurrence of any likeness of the historical injustices such as the experiments by the Nazi physicians, the Thalidomide case study, and the Tuskegee Syphilis study [9, 67]. They echo the view that COVID-19 “should not be viewed as an opening to opportunistically reduce participant protection” [67] (p. 9).

In the context of PHE, many study participants and communities could be vulnerable, desperate to participate in any research that offers some hope of therapy and hence the potential for exploitation can be heightened [7, 71]. Our reviewed articles emphasised ensuring that communities/societies facing PHEs adequately understand the rationale for the research being undertaken in their context and why it is important [59, 60, 62]. Respect for communities and participants is especially critical at this time; literature on previous PHEs or disasters can be drawn on to inform how to ensure fairness at every stages of the research and/or research intervention activities [2, 7, 19, 60]. For clinical trials, Folayan and colleagues proposed a comprehensive stakeholder engagement with interest groups who can positively or negatively impact how the proposed study or intervention would work, and emphasize the need for formative research to help identify these interest groups [62].

### Social science considerations: interdisciplinary composition of RECs during PHEs

Our findings show the critical role of formative research in facilitating the ethical conduct of research, including informing research design and contextually appropriate informed consent processes, as well as ways to strengthen rigour of the research and acceptable consent processes [59, 60]. Learning from previous experiences, there is now a marked emphasis, including within multilateral humanitarian institutions such as the WHO, in adequately incorporating social science into biomedical research [71].

In addition, insights from social scientists are important within the REC review processes, especially during PHEs, given the socio-behavioural aspects of the spread and containment of infectious diseases. As some commentators and contexts showed within the COVID-19 research context where community engagement and support for research was needed [67, 68], flexibility within RECs mechanisms is crucial, such as bigger, inclusive, interdisciplinary, and collaborative review teams [67]. This is important because of the drive to achieve a balance between a biomedical research focus, the promotion of ethical principles, and support for good health outcomes through the skills, competence and reach that members, especially social scientists, bring to the review of research during PHEs and beyond [43, 72, 73]. Social science research approaches contribute within research designs, as independent studies, enhancing contextual understandings of the people, participants, or targets of research (and interventions) [60, 65]. This is more crucial within environments riddled with mistrust of health practitioners and researchers in particular, as well as strong cultural beliefs and interpretations of and engagement with medical practices and modalities from the Global North [59, 60, 71, 74–76].

### Study limitations

An important limitation of this scoping review is that our search strategy was aimed to identify African experiences however, findings were largely limited to certain regions due to the available literature during our search timeframe. Also, while the diversity of focus within the articles reviewed demonstrates different components of the ethics review processes and frameworks required for ethical conduct of research during PHEs, many of the arguments made in this review are inferred. Essentially, we argued that authors’ emphasis on certain components of the ethical conduct of research implies a recommendation for what RECs should consider in their engagement with research protocols during the context of a PHE. Hence, a reading of some of the articles reviewed will point to discussions, for example, about the importance of stakeholder engagement and various aspects of informed consent application within the establishment and execution of studies during COVID-19 [58, 62]. While the components remain crucial for ethics review processes, they were not discussed in-depth by the authors in that sense, hence our inferential conclusions.

## Conclusion

This scoping review reveals that much more needs to be done around ethics review processes and procedures within Africa for better preparedness and response to emerging and future pandemics. This is needed so that timely context-relevant research can be undertaken but also in a manner that adequately recognises and reinforces the dignity of people in the quest to gain more understanding of diseases. This requires training and capacity building for REC members, reviewing the make-up and competencies of RECs to handle specific cases during PHEs, and for governments and study funders to make funds more flexible and durable to allow for training and more ingenious approaches to research review during public health emergencies.

Furthermore, drawing from the findings of this review, especially those around the dearth of literature on ethics review processes within Africa during PHEs, we recommend more documentation of the experiences linked to planning and implementation of ethics review processes. Donors can facilitate this by supporting the development of reflections on these experiences to be documented and duly published. Also, aligning with Bain and colleagues [61], deepening mechanisms that would ensure continued and stronger social science contributions not only within community engagement components but in the review processes would prove crucial for strengthening the review processes during PHEs.

## Data Availability

All the data generated or analysed during this scoping review have been included in this manuscript.
